# Heterochronic maturation of anatomical plugs for protecting the airway in rorqual whales (Balaenopteridae)

**DOI:** 10.1098/rsos.220459

**Published:** 2022-12-14

**Authors:** Henrik Lauridsen, Charlotte Bie Thøstesen, Christina Carøe Ejlskov Pedersen, Steffen Ringgaard, Mette Elstrup, Peter Rask Møller, Daniel Klingberg Johansson, Aage Kristian Olsen Alstrup

**Affiliations:** ^1^ Department of Clinical Medicine, Aarhus University, 8200 Aarhus N, Denmark; ^2^ The Fisheries and Maritime Museum, 6710 Esbjerg V, Denmark; ^3^ Department of Forensic Medicine, Aarhus University, 8200 Aarhus N, Denmark; ^4^ Department of Natural History, Museum of Southern Jutland, 6510 Gram, Denmark; ^5^ Natural History Museum of Denmark, University of Copenhagen, 2100 Copenhagen Ø, Denmark; ^6^ Norwegian College of Fishery Science, UiT – The Arctic University of Norway, 9037 Tromsø, Norway; ^7^ Department of Nuclear Medicine & PET, Aarhus University Hospital, 8200 Aarhus N, Denmark; ^8^ Department of Chemistry and Bioscience, Aalborg University, 9220 Aalborg Ø, Denmark

**Keywords:** lunge feeding, oral plug, nasal plug, development, magnetic resonance imaging, X-ray computed tomography imaging

## Abstract

Recently, a unique mechanism for protecting the airway during lunge feeding was discovered in rorqual whales (Balaenopteridae). This mechanism is based on an oral plug structure in the soft palate with similarities in musculo-fatty composition to the nasal plugs protecting the respiratory tract of rorquals from water entry and barotrauma during diving. As a follow-up, we present here a developmental series on fetal, prenatal, juvenile and adult specimens across five species of rorquals, showing differential maturation of the nasal and oral respiratory protection plugs. Nasal plugs are fully formed to serve an immediate crucial function at birth. By contrast, the soft palate remains muscular until the onset of solid food intake, where a musculo-fatty oral plug is developed.

## 1. Introduction

Rorquals, encompassing some of the largest animals on Earth, use an engulfment feeding method, lunge feeding, unique to these giants and a few other animals such as pelicans (*Pelecanus* spp.), gulper eels (*Eurypharynx pelecanoides* and *Saccopharynx* spp.) and potentially also extinct marine reptiles of the genus *Hupehsuchus* [1]. During a rorqual lunge feeding event, the mouth is opened to an approximately 80° gape during accelerated body speeds of approximately 2–5 m s^−1^ [2,3], thereby expanding the elastic mouth floor and engulfing a quantity of water and prey constituting more than 150% of the whales own body mass in large individuals [4]. This feeding mode exposes the mouth and associated structures to immense biomechanical forces [5]. In recent years, two unique anatomical structures, nasal and oral plugs, for protecting the upper airway in rorquals have been described [6,7]. Both plugs are musculo-fatty in composition in adult individuals to provide a tight seal in the relaxed state in the nasal cavities and at the oropharyngeal channel [6,7]. The nasal plugs are positioned in the nasal passages and consist of a rostrally positioned muscular portion, a fatty mid-portion and a caudally positioned tendon [6,7]. In the relaxed state, the fatty portion is drawn into the nasal passage by the tendon to provide a seal, thus opening the nasal cavities requires active withdrawal of the plug by the nasal plug muscles [6,7]. This is possible because it is the fatty portion that provides the seal, rather than the muscular portion which expands during contraction. The oral plug is positioned in the soft palate and can change position to seal off the caudal portion of the oral cavity during breathing or occlude the upper airway by taking up the nasopharyngeal space during swallowing [7]. Previous non-invasive magnetic resonance imaging of a fetal minke whale of unspecified size and fetal stage revealed somewhat developed nasal plugs before birth [6], whereas dissection of a fetal fin whale at approximately 46% birth length showed an incompletely developed oral plug [7]. This seeming heterochrony of the development of nasal and oral respiratory protection plugs in rorquals could indicate differential importance of these structures in early life and during nursing.

## 2. Methods

### 2.1. Specimen information

To investigate the fetal and juvenile maturation of the two types of respiratory protection plugs, we non-invasively imaged the upper airway on a series of early fetal (approx. 20% gestation time), mid-fetal (40–50% gestation time) and prenatal (approx. 90% gestation time) rorqual specimens (per cent gestation time based on total length of fetus and fetal characteristics such as disappearance of the teeth and appearance of baleen according to Roston *et al*. and Lanzetti *et al*. [8,9]). These were obtained from the preserved collection of mammals at the Natural History Museum of Denmark, University of Copenhagen and The Fisheries and Maritime Museum, Denmark. Additionally, we imaged the excised oral and nasal plugs of a beached adult rorqual to validate previous descriptions of muscle and fat distribution [6,7]. A full developmental series of a single rorqual species was not available, but because lunge feeding and the morphological adaptations involved in this feeding technique can be inferred to have evolved already in the shared common ancestor of the family Balaenopteridae [10,11], specimens of four species of rorquals were non-invasively imaged: early fetal blue whale *Balaenoptera musculus* (Linnaeus, 1758) catalogue no. NHMD-CN21, 201.5 mm total length (approx. 2.7% birth length), sampled in South Georgia in 1933, preserved in 70% v/v ethanol; mid-fetal blue whale catalogue no. NHMD-M08-1108, 938.7 mm total length (approx. 12.5% birth length), sampling year and location unknown, preserved in 70% v/v ethanol; early fetal fin whale *Balaenoptera physalus* (Linnaeus, 1758) catalogue no. NHMD-CN11, 248.1 mm total length (approx. 3.9% birth length), sampled in the Faroe Islands in 1897, preserved in 70% v/v ethanol; mid-fetal humpback whale *Megaptera novaeangliae* Borowski, 1781 catalogue no. NHMD-M08-1115, 795.9 mm total length (approx. 17.7% birth length), sampling year and location unknown, preserved in 70% v/v ethanol; prenatal minke whale *Balaenoptera acutorostrata* Lacépède, 1804 catalogue no. C427, 1870 mm total length (approx. 77.3% birth length), found by commercial fishermen freshly dead in the North Sea approximately 160 km west of the Danish town of Søndervig in October 2021, initially preserved in a frozen state and later thawed before imaging procedures; adult female minke whale (non-pregnant, at least one previous calf) catalogue no. NHMD-M08-1760, 7800 mm total length (109% of length at female maturity), beached at the Danish town of Thyborøn in the North Sea in May 2022, thoroughly dissected on land. Birth lengths are according to Lanzetti *et al*. [9]. Additionally, we photo-documented the presence/absence of the oral plug in two rorqual individuals estimated to be of weaning age: male sei whale *Balaenoptera borealis* Lesson, 1828 catalogue no. NHMD-M08-1695, 7700 mm total length (approx. 165% birth length, 56.2% adult male length), beached in Mariagerfjord approximately 3 km east of the Danish city of Hobro in December 2018, thoroughly dissected on land; female humpback whale catalogue no. NHMD-M08-1698, 8400 mm total length (187% birth length, 60% adult female length), found by commercial fishermen freshly in the sea of Kattegat approximately 250 m east of the Danish town of Skagen in October 2019, thoroughly dissected on land.

### 2.2. Imaging

On small specimens (*Balaenoptera musculus* catalogue no. NHMD-CN21 and *Balaenoptera physalus* catalogue no. NHMD-CN11) magnetic resonance imaging was performed using an Agilent 9.4 T system equipped with a 91 mm quadrature volume coil and using a three-dimensional gradient echo sequence with the following parameters: repetition time = 7 ms, echo time = 3.7 ms, flip angle = 30°, field-of-view = 70 × 70 × 70 mm^3^, spatial resolution 0.137 mm isotropic, number of averages = 40, acquisition time = 22 h.

On larger specimens (*Balaenoptera musculus* catalogue no. NHMD-M08-1108, *Megaptera novaeangliae* catalogue no. NHMD-M08-1115 and *Balaenoptera acutorostrata* catalogue no. C427), magnetic resonance imaging was performed using a Siemens Magnetom Skyra 3 T system equipped with a row of coils in the scanner table and two Siemens Body 18 and one Flex Large 4 surface coils using a three-dimensional spin echo sequence with the following parameters: repetition time = 2000 ms, echo time = 130 ms, refocusing flip angle = 120̊, field-of-view = 461 × 173 × 14 mm^3^, spatial resolution 0.45 mm isotropic, number of averages = 4 and acquisition time = 2 h.

On all specimens, X-ray computed tomography was performed using a Canon Aquilion Prime SP system with a range of parameters depending on sample size: X-ray tube voltage = 120 kVp, X-ray tube current = 140–200 mA, integration time = 300–1000 ms, field-of-view = 102 × 102 × 261–539 × 539 × 1902 mm^3^, spatial resolution = 0.2–1 mm isotropic and convolution kernel = FC18, acquisition time = 60 s per scan.

Image J (version 1.50e) and OsiriX DICOM Viewer were used for image viewing and reslicing.

## 3. Results

**Figure 1.**
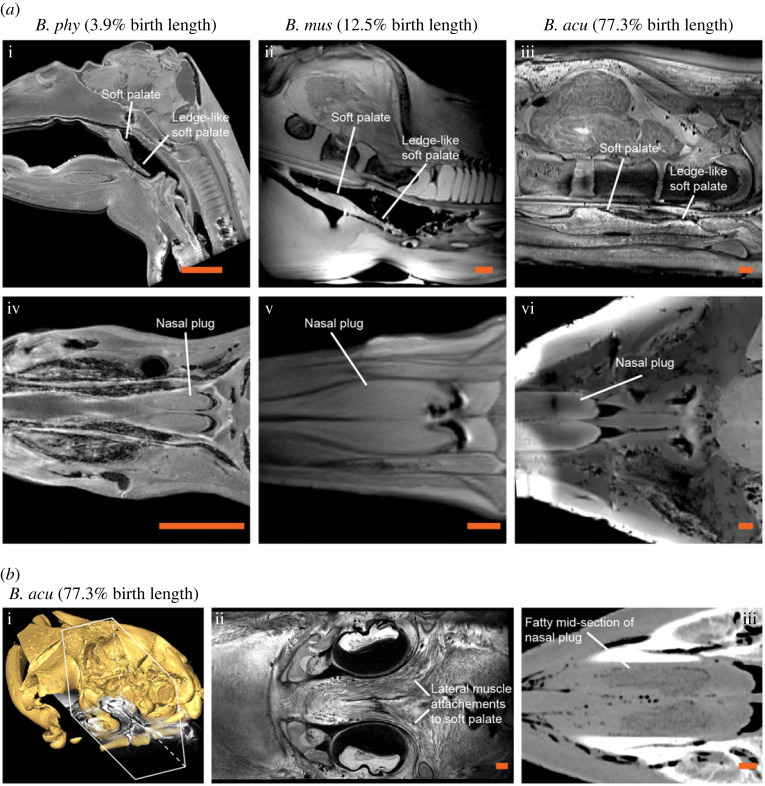
The developing soft palate and nasal plugs in rorquals. (*a*) Sagittal (i–iii) and coronal (iv–vi) magnetic resonance imaging slices through the head of an early fetal fin whale (*Balaenoptera physalus*, *B. phy*), a mid-fetal blue whale (*Balaenoptera musculus*, *B. mus*) and a prenatal minke whale (*Balaenoptera acutorostrata*, *B. acu*) showing the developing soft palate without tissue contrasts as indicative of a musculo-fatty oral plug (i–iii) and increasingly mature nasal plugs (iv–vi). (*b*) On (i), posterolateral view of digitally cropped (white box) minke whale skull from X-ray computed tomography surface reconstruction with co-registered coronal magnetic resonance imaging slice through the soft palate. In (ii), coronal magnetic resonance imaging slice (same as on (i)) through the soft palate showing muscle fibre attachment to the lateral sides of the nasopharynx. On (iii), coronal X-ray computed tomography slice through the nasal plugs clearly showing hypointense fatty mid-sections of the nasal plugs already formed. Scale bars represent 10 mm on all images.

**Figure 2.**
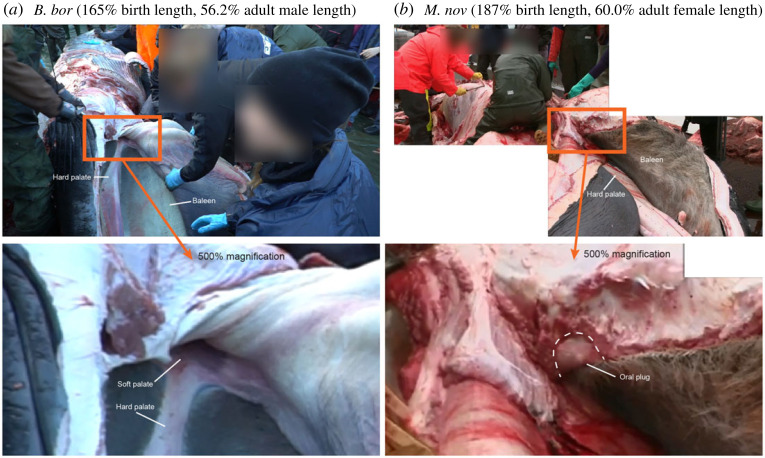
Oral plugs in rorquals at the age of weaning. (*a*) Video frame of a young sei whale (*Balaenoptera borealis*, *B. bor*) calf of estimated pre-weaning age in a supine position with the oral cavity exposed though dissection. The tongue is in the process of being excised. The rostral portion of the soft palate is visible revealing the lack of a fully formed musculo-fatty oral plug. Video by The Fisheries and Maritime Museum, Denmark. (*b*) Two combined video frames of a young humpback whale (*Megaptera novaeangliae*, *M. nov*) calf estimated to be of weaning or recently weaned age in a supine position with the oral cavity exposed through dissection. The tip of the oral plug can be observed at the caudal portion of the oral cavity. Video by Stefan Kjærgaard—TV2 Nord.

**Figure 3.**
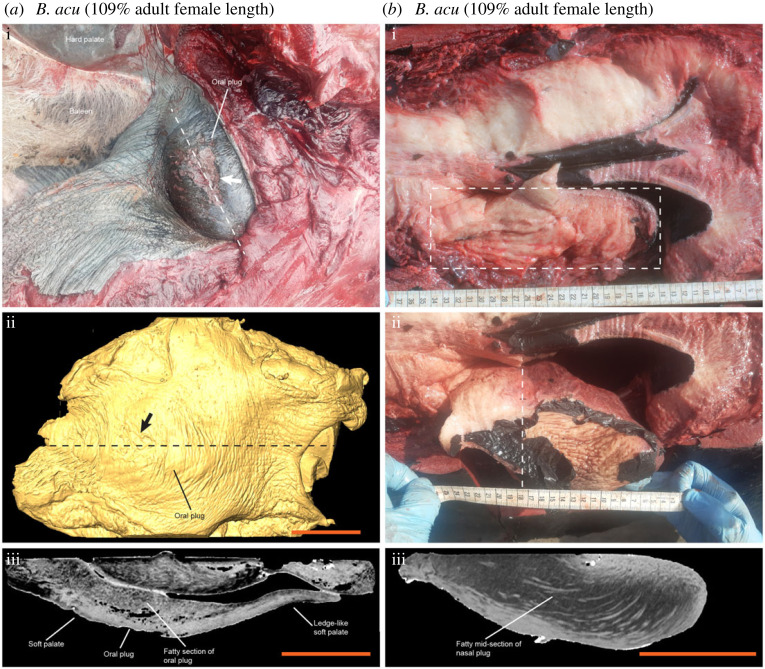
Oral and nasal plugs in adult minke whale. (*a*) On (i), photo of oral plug in adult minke whale (*Balaenoptera acutorostrata*, *B. acu*). A recognizable abrasion mark is labelled with white arrow. This photo from the beached minke whale lying on its back has been rotated so the hard palate appears at the top of the image as it would if the whale was approximately right-side up. In (ii), X-ray computed tomography surface reconstruction of excised oral plug with abrasion mark labelled with a black arrow. At (iii), sagittal X-ray computed tomography slice through the oral plug along the dashed lines in (i) and (iii) images. A hypointense section of the soft palate reveals a fatty portion of this structure in a mature rorqual. (*b*) On (i) and in (ii), photos of nasal plugs in place (i) and with the left nasal plug partly resected (ii) in adult minke whale. At (iii), coronal X-ray computed tomography slice through the left nasal plug along the dashed plane and lines in (i) and (ii) images. A hypointense mid-section of the nasal plug reveals at fatty portion of this structure in a mature rorqual. Scale bars represent 100 mm on all images.

Both the developing soft palate and nasal plugs were easily distinguishable in all stages of fetal development in all studied specimens (figure 1*a*; electronic supplementary material, figure S1 and video S1). The soft palate could be separated into a rostral thick section and a caudal ledge-like section (figure 1*a*; electronic supplementary material, figure S1a). However, no fatty composition indicative of an oral plug was observable at the thick section of the soft palate in any of the fetal or prenatal stages (figure 1*a*; electronic supplementary material, figure S1a). At the late prenatal stage of the minke whale, clear lateral muscle fibres from the soft palate could be observed to attach to the lateral sides of the nasopharynx (figure 1*b*i,ii). No circular muscle fibres indicative of a sphincter were observable at the base of the nasopharynx (electronic supplementary material, figure S1a). The fatty composition of the nasal plugs could only be observed at the latest prenatal stage of the minke whale (figure 1*a*iii and 1*b*iii). Dissection of a pale-fleshed sei whale calf which was estimated to have been nursing at the time of death based on length, the lack of any stomach indications of solid food intake and the visibly low muscular myoglobin content [12], revealed no clear musculo-fatty oral plug but rather a reddish and smooth soft palate as seen in fetal specimens (figure 2*a*). However, an oral plug structure could be identified in a young humpback whale, which was estimated to be slightly older than the sei whale due to the appearance of red flesh and indications of solid food intake (figure 2*b*). Both the oral and nasal plugs of an adult minke whale contained a fatty section (figure 3).

## 4. Discussion

Both the nasal and the oral plugs are believed to provide protective functions in the upper respiratory tract in rorquals, but the circumstances under which these plugs act are different [6,7]. The nasal plugs are probably crucial to avoid water entry when submerged and to alleviate the risk of developing barotrauma by gradually reducing the overall incompressible air-filled space during diving by sliding into the nasal cavity [6]. A nasal plug entirely composed of muscular tissue would not serve this function well, since the cross-sectional area of muscle increases during contraction, and thus purely muscular nasal plugs would hardly be able to slide in and out of the nasal cavity [6]. Since rorqual calves undertake diving behaviour soon after birth to follow their mothers [13], it is not a surprise that fully developed nasal plugs with fatty mid-sections are already developed prenatally (figure 1*b*iii), suggesting that blowhole margins are not sufficient to support a nasal seal even at the moderate depths of calf diving.

The oral plug of rorquals is yet undescribed in other mysticete species which may suggest that its function is strictly related to lunge feeding. In our developmental series, we did not observe an apparently fully developed musculo-fatty oral plug until late or post weaning (figure 2*b* and figure 3). Until this point, the soft palate consists purely of muscular tissue connected to the lateral side of the nasopharynx. This suggests that a dedicated oral plug is not needed during nursing and that a muscular soft palate can create a sufficient oropharyngeal seal at this stage. The muscular suspension of the thick section of the soft palate that eventually develops into the oral plug (figure 1*b*i,ii) supports the recently suggested mechanism that the soft palate can be elevated dorsocaudally during swallowing, thereby displacing the oral plug (once formed) from the oropharyngeal inlet [7].

In summary, our developmental series on eight specimens of five species of rorquals reveal heterochronic maturation of the respiratory protection plugs. Although we recognize the potential for interspecific variation in a developmental series consisting of different species, the overall conclusion is that nasal plugs in rorquals serve an immediate crucial function at birth, whereas oral plugs are not needed before the onset of solid food intake.

## Data Availability

Magnetic resonance imaging and X-ray computed tomography data are available on MorphoSource (https://www.morphosource.org/) project no. 000429230. The data are provided in the electronic supplementary material [14].
